# Epidemiology, prehospital care and outcomes of patients arriving by ambulance with dyspnoea: an observational study

**DOI:** 10.1186/s13049-016-0305-5

**Published:** 2016-09-22

**Authors:** Anne Maree Kelly, Anna Holdgate, Gerben Keijzers, Sharon Klim, Colin A. Graham, Simon Craig, Win Sen Kuan, Peter Jones, Charles Lawoko, Said Laribi, AANZDEM study group, Richard McNulty, David Lord Cowell, Anna Holdgate, Nitin Jain, Tracey De Villecourt, Kendall Lee, Dane Chalkley, Lydia Lozzi, Stephen Edward Asha, Martin Duffy, Gina Watkins, David Rosengren, Jae Thone, Shane Martin, Ulrich Orda, Ogilvie Thom, Frances Kinnear, Michael Watson, Rob Eley, Alison Ryan, Douglas Gordon Morel, Jeremy Furyk, Richard D. B. Smith, Michelle Grummisch, Robert Meek, Pamela Rosengarten, Barry Chan, Helen Haythorne, Peter Archer, Simon Craig, Kathryn Wilson, Jonathan Knott, Peter Ritchie, Michael Bryant, Stephen MacDonald, Mlungisi Mahlangu, Peter Jones, Michael Scott, Thomas Cheri, Mai Nguyen, Colin A. Graham, Melvin S. Y. Chor, Chi Pang Wong, Tai Wai Wong, Ling-Pong Leung, Chan Ka Man, Ismail Mohd Saiboon, Nik Hisamuddin Rahman, Wee Yee Lee, Francis Chun Yue Lee, Shaun E. Goh, Win Sen Kuan, Sharon Klim, Kerrie Russell, Anne-Maree Kelly, Gerben Keijzers, Charles Lawoko

**Affiliations:** 1 Joseph Epstein Centre for Emergency Medicine Research @ Western Health, Sunshine, Australia; 2 School of Medicine-Western Clinical School, The University of Melbourne, Parkville, Australia; 3 Department of Emergency Medicine, Liverpool Hospital, University of New South Wales (Southwest Clinical School), Sydney, Australia; 4 Department of Emergency Medicine, Gold Coast University Hospital, Gold Coast, QLD Australia; 5 School of Medicine, Bond University, Gold Coast, QLD Australia; 6 School of Medicine, Griffith University, Gold Coast, QLD Australia; 7 Prince of Wales Hospital, Chinese University of Hong Kong, Shatin, Hong Kong SAR; 8 Emergency Department, Monash Medical Centre, Clayton, Australia; 9 School of Clinical Sciences, Monash University, Clayton, Australia; 10 Murdoch Children’s Research Institute, Parkville, Australia; 11 Emergency Medicine Department, National University Health System, Singapore, Singapore; 12 Department of Surgery, Yong Loo Lin School of Medicine, National University of Singapore, Singapore, Singapore; 13 Department of Emergency Medicine, Auckland City Hospital, Auckland, New Zealand; 14 Statistical Consulting Service, Graduate Research Centre, Victoria University, Footscray, Australia; 15 Emergency Medicine Department, Tours University Hospital, 37044 Tours, France

**Keywords:** Dyspnoea, Ambulance, Emergency department, Epidemiology

## Abstract

**Background:**

This study aimed to determine epidemiology and outcome for patients presenting to emergency departments (ED) with shortness of breath who were transported by ambulance.

**Methods:**

This was a planned sub-study of a prospective, interrupted time series cohort study conducted at three time points in 2014 and which included consecutive adult patients presenting to the ED with dyspnoea as a main symptom. For this sub-study, additional inclusion criteria were presentation to an ED in Australia or New Zealand and transport by ambulance. The primary outcomes of interest are the epidemiology and outcome of these patients. Analysis was by descriptive statistics and comparisons of proportions.

**Results:**

One thousand seven patients met inclusion criteria. Median age was 74 years (IQR 61-68) and 46.1 % were male. There was a high rate of co-morbidity and chronic medication use. The most common ED diagnoses were lower respiratory tract infection (including pneumonia, 22.7 %), cardiac failure (20.5%) and exacerbation of chronic obstructive pulmonary disease (19.7 %). ED disposition was hospital admission (including ICU) for 76.4 %, ICU admission for 5.6 % and death in ED in 0.9 %. Overall in-hospital mortality among admitted patients was 6.5 %.

**Discussion:**

Patients transported by ambulance with shortness of breath make up a significant proportion of ambulance caseload and have high comorbidity and high hospital admission rate. In this study, >60 % were accounted for by patients with heart failure, lower respiratory tract infection or COPD, but there were a wide range of diagnoses. This has implications for service planning, models of care and paramedic training.

**Conclusion:**

This study shows that patients transported to hospital by ambulance with shortness of breath are a complex and seriously ill group with a broad range of diagnoses. Understanding the characteristics of these patients, the range of diagnoses and their outcome can help inform training and planning of services.

## Background

Despite respiratory distress being a common reason for ambulance transfer to hospital [1], little is known about the epidemiology and outcome of this important patient group. The only previous study from the United States examined patients categorised by emergency medical service (EMS) personnel as having respiratory distress and reported the common diagnoses as being heart failure, pneumonia, chronic obstructive pulmonary disease (COPD) and respiratory failure, a 50 % admission rate and 10 % mortality among patients who were admitted to hospital [1]. There is no similar published data reported for Europe, Australasia or other regions.

Understanding the characteristics of these patients, the range of diagnoses and their outcome are important for understanding the challenges facing prehospital clinicians and for planning training and services.

This study aimed to describe the epidemiology and outcome for patients presenting to emergency departments (ED) with shortness of breath who were transported by ambulance.

## Methods

### Study design and governance

This is a planned sub-study of a prospective, interrupted time series cohort study conducted in EDs in Australia, New Zealand, Singapore, Hong Kong and Malaysia the methodology of which has been previously published [2]. The project was overseen by a steering committee made up of researchers from across Australia, New Zealand, Europe, Singapore and Hong Kong.

### Site selection and participation

For the parent study, EDs were eligible to participate if they were an accredited ED according to local national criteria. Participation was by an expression of interest process. Directors of eligible EDs were contacted by email with an outline of the project and invited to participate.

This planned sub-study included patients presenting to an Australian or New Zealand ED by ambulance. The South East Asian sites were not included as they have markedly different prehospital care systems, in particular there are major differences in structures, training and in treatments that prehospital clinicians can administer. In Australia and New Zealand, paramedics are trained via a university degree course. Both also have a second tier of paramedics with advanced skills and treatment options (intensive care paramedics) based on additional training and significant field experience.

### Patient selection and data collection

Eligible patients were consecutive adult patients presenting with dyspnoea as a main symptom at ED presentation attending the ED during the three 72-h study periods (13–16 May 2014; 12–15 August 2014; 14–17 October 2014). These dates were chosen to represent different seasons (autumn, winter and spring) in the region. Summer was not included due to funding limitations. The parent study used a specifically designed data collection instrument and data dictionary that were developed by an iterative process by the steering committee. The data form was piloted on a small sample of cases not in the study period for validation. Local data collectors were instructed that dyspnoea was considered a main symptom if it was listed as a symptom at presentation or triage (systems varies slightly in how patient reception occurred).

Data was collected onto the validated data form by local clinician-investigators; nurses or doctors. Local data collectors were instructed to contact the co-ordinating centre by phone if they had any queries regarding data collection processes or data definitions. Data was then entered as de-identified data into a password-secured central study database managed by the Clinical Informatics and Data Management Unit, Faculty of Medicine, Nursing and Health Sciences, Monash University. Data collected included patient characteristics, co-morbidities, mode of arrival, usual medications, pre-hospital treatment as documented in ED clinical records, initial assessment (clinical assessment and vital signs), investigations performed in ED (laboratory tests, electrocardiogram (ECG), imaging, etc.) and results, treatment in the ED, ED diagnosis (diagnosis at conclusion of ED phase of care), disposition from ED, in-hospital outcome and final hospital diagnosis.

### Outcomes of interest and analysis

The primary outcomes of interest are the epidemiology and outcome of patients presenting to ED by ambulance with dyspnoea.

Analysis is by descriptive statistics. A formal sample size calculation was not performed as this is largely a descriptive study, however it was anticipated that data on >1000 patients would be eligible.

### Human research ethics approvals

HREC approval was obtained for all sites according to local requirements.

## Results

In Australia and New Zealand there were 37 participating sites – four in New Zealand and 33 in Australia (10 Queensland, 11, New South Wales, 10 Victoria and 2 Western Australia). The parent study enrolled 1957 patients, 1007 of whom arrived by ambulance (51.5 %, 95 % CI 49.3–53.7 %) comprising the study sample.

Characteristics of patients and outcome are shown in Table 1, comparing patients who arrived by ambulance with patients who did not. Patients who arrived by ambulance were significantly older, have more co-morbidity and were more likely to be taking cardiorespiratory medications. They were also more likely to require hospital admission and admission to ICU and had a higher mortality.

**Table 1:** Baseline characteristics and outcome

Variable	Arrival by ambulance (*N* = 1007)	Missing data (N)	Did not arrive by ambulance (*N* = 906)	Missing data (N)	Significance
Demographics					
Age (years, median, IQR)	74, 61–84	0	60, 40–75	0	*p* < 0.001
Age >60 years (N, %, 95 % CI)	764, 75.9 % (73.1–78.4 %)	0	447, 49.3 % (46.1–52.6 %)	0	*p* < 0.001
Gender (male, N, %, 95 % CI)	464, 46.1 % (43–49.2 %)	0	412, 45.5 % (42.3–48.7 %)	0	0.82
Co-morbidities (N, %, 95 % CI)					
Hypertension	538, 53.6 % (50.5–56.7 %)	3 (0.3 %)	337, 37.6 % (34.5–40.8 %)	10 (1.1 %)	<0.001
Dyslipidaemia	368, 36.8 % (33.8–39.8 %)	6 (0.6 %)	208, 23.9 % (20.6–26.2 %)	13 (1.4 %)	<0.001
COPD	364, 36.4 % (33.5–39.5 %)	8 (0.8 %)	167, 18.7 % (16.3–21.4 %)	13 (1.4 %)	<0.001
Ischaemic heart disease	308, 30.7 % (28–33.7 %)	5 (0.5 %)	147, 16.4 % (14.1–19 %)	11 (1.2 %)	<0.001
Heart failure	262, 26.2 % (23.5–29 %)	5 (0.5 %)	126, 14.1 % (12–16.5 %)	12 (1.3 %)	<0.001
Diabetes	257, 25.7 % (23–28.4 %)	5 (0.5 %)	169, 18.9 % (16.5–21.6 %)	13 (1.4 %)	<0.001
Atrial fibrillation	222, 22.2 % (19.7–24.8 %)	5 (0.5 %)	134, 15 % (12.8–17.5 %)	14 (1.6 %)	<0.001
Asthma	200, 20 % (17.6–22.6 %)	6 (0.6 %)	245, 27.5 % (24.7–30.5 %)	12 (1.3 %)	<0.001
Chronic renal disease	166, 16.6 % (14.4–19.1 %)	8 (0.8 %)	94, 10.5 % (8.7–12.7 %)	12 (1.3 %)	<0.001
Active smoker	151, 15.1 % (13–17.5 %)	8 (0.8 %)	147, 16.5 % (14.2–19.1 %)	14 (1.6 %)	0.45
Active malignancy	91, 9.1 % (7.5–11.1 %)	8 (0.8 %)	72, 8.1 % (6.5–10 %)	14 (1.6 %)	0.47
Previous pulmonary embolism	51, 5.1 % (3.9–6.7 %)	8 (0.8 %)	32, 3.6 % (2.6–5 %)	15 (1.7 %)	0.14
Regular/Usual medications (N, %, 95 % CI)					
Inhaled beta-agonists	403, 40.3 % (37.3–43.3 %)	6 (0.6 %)	319, 35.7 % (32.6–38.9 %)	12 (1.3 %)	0.05
Statins	403, 40.2 % (37.2–43.3 %)	4 (0.4 %)	227, 25.4 % (22.6–28.3 %)	11 (1.2 %)	<0.001
Angiotensin converting enzyme inhibitors/ similar	371, 37 % (34.1–40 %)	4 (0.4 %)	255, 28.6 % (25.7–31.6 %)	13 (1.4 %)	<0.001
Diuretic	347, 34.6 % (31.7–37.6 %)	4 (0.4 %)	173, 19.4 % (16.9–22.1 %)	14 (1.6 %)	<0.001
Aspirin	304, 30.3 % (27.6–33.2 %)	4 (0.4 %)	154, 17.2 % (14.9–19.8 %)	11 (1.2 %)	<0.001
Beta-blocker	279, 27.8 % (25.1–30.7 %)	4 (0.4 %)	170, 19 % (16.6–21.7 %)	12 (1.3 %)	<0.001
Calcium channel blocker	186, 18.6 % (16.3–21.1 %)	6 (0.6 %)	113, 12.7 % (10.7–15 %)	14 (1.6 %)	<0.001
Long acting anticoagulant	171, 17.1 % (14.9–19.6 %)	7 (0.7 %)	111, 12.4 % (10.4–14.8 %)	14 (1.6 %)	0.006
Oral corticosteroid	129, 12.9 % (11–15.1 %)	7 (0.8 %)	65, 7.3 % (5.8–9.2 %)	15 (1.7 %)	<0.001
Nitrate	111, 11.1 % (9.3–13.2 %)	8 (0.8 %)	44, 4.9 % (3.7–6.6 %)	16 (1.8 %)	<0.001
Clopidogrel	110, 11.0 % (9.2–13.1 %)	6 (0.6 %)	58, 6.5 % (5.1–8.3 %)	14 (1.6 %)	<0.001
Insulin	81, 8.1 % (6.6–9.9 %)	6 (0.6 %)	56, 6.3 % (4.9–8.1 %)	14 (1.6 %)	0.15
Cardiac glycoside	78, 7.8 % (6.3–9.6 %)	7 (0.7 %)	39, 4.4 % (3.2–5.9 %)	15 (1.5 %)	0.003
Home oxygen	67, 6.7 % (5.3–8.4 %)	6 (0.6 %)	24, 2.7 % (1.8–4 %)	15 (1.7 %)	<0.001
Clinical features^b^					
Duration of symptoms (days, median, IQR)	2, 1–5	36 (3.6 %)	3, 1–7	42 (4.6 %)	<0.001
Respiratory rate (mean, SD)	25 (8)	18 (1.8 %)	22 (6)	35 (3.9 %)	<0.001
O2 Saturation on air (mean, SD)	92.5 (7)	250^a^ (24.8 %)	94.9 (6)	53 (5.9 %)	<0.001
O2 saturation <90 % (N, %)	196, 19.9 % (17.5–22.5 %)	20 (2 %)	76, 8.6 % (6.9–10.6 %)	18 (2 %)	<0.001
Systolic BP <100 mmHg (N, %)	56, 5.9 % (4.6–7.5 %)	17 (1.7 %)	40, 4.7 % (3.5–6.3 %)	51 (5.6 %)	0.40
Systolic BP >180 mmHg (N, %)	71, 7.2 % (5.7–9 %)	17 (1.7 %)	36, 4.2 % (3.1–5.8 %)	51 (5.6 %)	0.009
Heart rate >100 (N, %)	390, 39.6 % (36.6–42.7 %)	22 (2.2 %)	264, 30 % (27.1–33.1 %)	27 (3 %)	<0.001
GCS (mean, SD)	15 (1)	77 (7.7 %)	15 (0.5)	115 (12.7 %)	<0.001
Admitted to ward (ward, ICU or transfer for admission)	769, 76.5 % (73.8–79 %)	2 (0.2 %)	468, 46.6 % (43.5–49.7 %)	1 (0.1 %)	<0.001
Admitted to ICU	56, 5.6 % (4.3–7.2 %)	2 (0.2 %)	17, 1.9 % (1.2–3 %)	6 (0.6 %)	<0.001
Died in ED	9, 0.9 % (0.5–1.7 %)	2 (0.2 %)	2, 0.2 % (0.06–0.7 %)	0	0.10
In-hospital mortality for admitted patients	50, 6.5 % (5–8.5 %)	0	9, 1.9 % (1–3.6 %)	0	<0.001
Length of stay for admitted patients	5, 3–8	0	5, 3–8	0	0.66

^a^Most remaining patients were on oxygen at ED arrival

^b^taken at ED arrival

The most common ED diagnoses were lower respiratory tract infection (including pneumonia, 22.7 %), cardiac failure (20.5 %) and exacerbation of COPD (19.7 %). ED disposition was hospital admission (including ICU) for 76.4 %, ICU admission for 5.6 % and death in ED in 0.9 %. Overall in-hospital mortality among admitted patients was 6.5 %. Prehospital treatment and ED diagnosis are summarised in Table 2.

**Table 2:** Summary of pre-hospital treatment, ED diagnosis and final hospital diagnosis

Variable	Result	Missing data
Pre-hospital treatments (N, %, 95 % CI)		
Oxygen	585, 58.1 % (55.4–61.5 %)	8 (0.8 %)
Inhaled beta-agonist	216, 21.4 % (18.9–24 %)	15 (1.5 %)
Nitrates	128, 12.9 % (10.9–15.1 %)	12 (1.2 %)
Aspirin	105, 10.6 % (8.8–12.7 %)	15 (1.5 %)
Inhaled anticholinergic	87, 9 % (7.4–11 %)	43 (4.3 %)
IV beta-agonist	26, 2.6 % (1.8–3.8 %)	17 (1.7 %)
Corticosteroid	26, 2.6 % (1.8–3.8 %)	17 (1.7 %)
Opiate (morphine or fentanyl)	26, 2.6 % (1.8–3.8 %)	16 (1.6 %)
IV fluids	20, 2.1 % (1.3–3.2 %)	43 (4.3 %)
Non-invasive ventilation	18, 1.8 % (1.2–2.9 %)	16 (1.6 %)
Diuretic	18, 1.8 % (1.2–2.9 %)	16 (1.6 %)
Adrenaline	9, 0.9 % (0.5–1.7 %)	16 (1.6 %)
Endotracheal intubation	0, 0 % (0–0.4 %)	16 (1.6 %)
Thrombolysis	0, 0 % (0–0.4 %)	16 (1.6 %)
ED diagnosis (N, %, 95 % CI)		
Lower respiratory tract infection (including pneumonia)	229, 22.7 % (20.2–25.4 %)	0
Cardiac failure	204, 20.3 % (17.9–22.9 %)	0
COPD	198, 19.7 % (17.3–22.2 %)	0
Asthma	79, 7.9 % (6.3–9.7 %)	0
Acute coronary syndrome	28, 2.8 % (1.9–4 %)	0
Atrial fibrillation	27, 2.7 % (1.9–3.9 %)	0
Hyperventilation	16, 1.6 % (1–2.6 %)	0
Pleural effusion	15, 1.5 % (0.9–2.4 %)	0
Malignancy	14, 1.4 % (0.8–2.3 %)	0
Pulmonary embolism	12, 1.2 % (0.7–2.1 %)	0
Pneumothorax	4, 0.4 % (0.2–1 %)	0
Other/ unclear	181, 18 % (15.7–20.5 %)	0
Final hospital diagnosis (N, %, 95 % CI)		
Lower respiratory tract infection (including pneumonia)	236, 23.4 % (20.9–26.2 %)	0
Cardiac failure	186, 18.5 % (16.2–21 %)	0
COPD	197. 19.6 % (17.2–22.1 %)	0
Asthma	76, 7.6 % (6.1–9.3 %)	0
Acute coronary syndrome	29, 2.9 % (2–4.1 %)	0
Atrial fibrillation	21, 2.1 % (1.4–3.2 %)	0
Hyperventilation	15, 1.5 % (0.9–2.4 %)	0
Pleural effusion	11, 1.1 % (0.6–1.9 %)	0
Malignancy	18, 1.8 % (1.1–2.8 %)	0
Pulmonary embolism	9, 0.9 % (0.5–1.7 %)	0
Pneumothorax	4, 0.4 % (0.2–1 %)	0
Other/ unclear	205, 20.4 % (18–23 %)	0

## Discussion

Our data confirm that patients transported to hospital by ambulance with shortness of breath are a complex and seriously ill group. They are significantly older with more co-morbidity and chronic medication use than similar patients who do not arrive at ED by ambulance. Consistent with this is the high rate of hospital admission and significant in-hospital mortality-significantly more than non-ambulance patients. They also make up a substantial proportion of ambulance caseload, with previous research suggesting that approximately 12 % of cases in a United States study were for patients with respiratory distress [1]. A Swiss study of 10 year trends in prehospital care reported that approximately 6 % of cases have a main symptom of dyspnoea [3] and a Chinese study estimated the proportion as 8 % [4].

Three diagnoses accounted for >60 % of cases (heart failure, COPD and lower respiratory tract infection). This points to the importance of chronic disease management in reducing exacerbations of these conditions which may reduce the need for ambulance transport and hospital-based treatment. The ‘other’ group was surprisingly large; a reminder of the diversity of causes for dyspnoea. Included among that group were abdominal diagnoses such as pancreatitis, bowel obstruction, biliary colic and gastritis, neurological diagnoses such as motor neurone disease and vocal cord dysfunction, metabolic causes such as dehydration and hyponatraemia and a range of psychiatric diagnoses. This concurs with the findings of the study of Prekker et al. that reported that 40–47 % of patients had a discharge diagnosis not related to respiratory disease [1]. Taken together, this data reinforces the challenge faced by paramedics in accurately identifying the cause of dyspnoea without access to diagnostic tests or detailed past medical history.

The diagnoses accounting for most of the patients are similar between this study and the previous United States study [1]. Our study however had a higher admission rate (76.4 % vs. 51.1 %), a lower rate of ICU admission (5.6 % vs. 15.6 %) and lower in-hospital mortality among admitted patients (6.5 % vs. 10 %). Our study also found a lower rate of prehospital endotracheal intubation (0 % vs. >5 %). Without accurate prehospital clinical data for both studies, it is difficult to determine the factors that might explain the differences observed. Although not a direct comparison, the prehospital vital signs reported by Prekker et al. [1] and the ED arrival vital signs of this study report similar respiratory rates, Glasgow Coma Scores, oxygen saturations on air and rates of significant hypotension (BP <100 mmHg). There are significant differences in the proportion of patients with significant hypertension (BP >180 mmHg, US study higher, *p* < 0.001) and tachycardia (pulse rate >100, Australasia higher, *p* < 0.001). We consider it unlikely that these differences in vital sign distributions represent major differences in illness severity.

We found similar lengths of hospital stay despite the lower ICU admission rate (5 days vs. 4 days). This may reflect how ICUs are defined and used in the different countries rather than a true difference in disease severity. There may also be differences in how ambulance services are used by their communities, in cost and in the treatment modalities available to paramedics. An Indian study of patients with a chief complaint of dyspnoea attended by prehospital services reported a 72 h mortality of 27 % [5]. While confirming that shortness of breath is a high risk symptom for mortality, major differences between the Indian health system and its prehospital services and the others with available data make further comparison impossible.

The rate of non-invasive ventilation in this study was low (1.8 %). This treatment, specifically CPAP, has been shown to reduce mortality and intubation rates in patients with acute respiratory failure in the pre-hospital setting [6]. The low rate may be accounted for by this treatment only recently having been commenced by ambulance services in a number of the regions studied.

There are a range of models of prehospital services in use around the world. In Europe a number of countries have a prehospital response that includes a doctor who may have a range of speciality backgrounds, including anaesthesiology, emergency and ICU. In other parts of the world, particularly developing countries, prehospital services can be simply transport services with limited training and little ability to provide therapy other than first aid. The model used in Australasia is one of university trained paramedics (usually a 3 year course) who are able to provide a range of treatments including intravenous therapy, analgesia and bronchodilators according to protocols. It is also a two-tiered system with intensive care paramedics who have additional training and experience being able to provide advanced treatments such as endotracheal intubation. Although these service models are quite different, many aspects of our findings are generalizable particularly to developed countries. These include the age, co-morbidity and medication profiles of patients, the range of likely diagnoses and the estimated admission rate. Also generalizable are the opportunities for prevention through better chronic disease management and development of alternatives to ED presentation and hospital admission such as outreach services. Direct comparisons of prehospital service models and impacts on outcomes are scarce. Christenszen et al. reported a before and after study comparing outcomes with a standard ambulance service (basic life support and very limited non-parenteral treatment options) and a service with one response vehicle also staffed by an anaesthesiologist (mobile emergency care unit, MECU) [7]. Twenty seven percent of patients were treated by the MECU. That study reported a difference between periods in the proportion of patients transported to hospital (a reduction of 5 %) but no change in overall mortality. There however was a reduction in mortality for the subgroups with acute myocardial infection or respiratory disease. There is no high quality evidence comparing outcomes for various prehospital service models or cost benefit analysis.

The data in our study has potential implications for paramedic training and planning of pre-hospital services in similar prehospital services. It emphasises the broad range of causes that needs to be considered for a common symptom such as dyspnoea and the importance of assessment skills to be able to differentiate between possible causes. Previous research has shown that trained paramedics have good accuracy for the identification of the cause of dyspnoea as cardiac, respiratory or other with a percent agreement with the ED physician diagnosis of 81 % [8]. It may also prompt services to revise their treatment protocols for some of the more common conditions. There may also be implications for service planning. With an ageing population and increasing prevalence of chronic disease, there may be caseload implications to be considered in planning future services. There may also be opportunities to develop alternatives to traditional pre-hospital to ED pathways in partnership with chronic disease management programs. The data also informs ED with respect to service planning and bed demand as of the order of 75 % of ambulance patients with dyspnoea require hospital admission.

Our study has some limitations that should be considered when interpreting its results. The study sites were located in Australasia and may not be generalizable to other regions, largely based on paramedic training and role and variations in ambulance staffing (e.g. doctor on ambulances in some European countries). The sample reflects patients with dyspnoea who presented to ED by ambulance, the pre-hospital care system available in the study countries. It is possible that some patients who complained of dyspnoea were treated in-situ by ambulance services and were not transported to ED. It is not possible to quantify how many patients this applied to but the number would be expected to be small as it was usual practice in the study countries to transport patients with significant symptoms. Patients were identified for inclusion based on their presenting/ triage complaints. It is possible that error in documentation missed some patients and that some patients with dyspnoea did not report it until later in the clinical encounter. We do not consider these likely to have added systematic bias. There is a modest amount of missing data for some data items that may have influenced the results.

## Conclusion

This study shows that patients transported to hospital by ambulance with shortness of breath are a complex and seriously ill group with a broad range of diagnoses. Understanding the characteristics of these patients, the range of diagnoses and their outcome should inform training and planning of services.

## Abbreviations

CI: Confidence interval
COPD: Chronic obstructive pulmonary disease
ED: Emergency department(s)
EMS: Emergency medical service
ICU: Intensive care unit
IQR: Interquartile range
N: Number
STROBE: Strengthening the reporting of observational studies in epidemiology
